# Biomechanical duality of fracture healing captured using virtual mechanical testing and validated in ovine bones

**DOI:** 10.1038/s41598-022-06267-8

**Published:** 2022-02-15

**Authors:** Brendan Inglis, Peter Schwarzenberg, Karina Klein, Brigitte von Rechenberg, Salim Darwiche, Hannah L. Dailey

**Affiliations:** 1 Department of Mechanical Engineering and Mechanics, Lehigh University, Bethlehem, PA 18015 USA; 2 Musculoskeletal Research Unit (MSRU), Vetsuisse Faculty, University of Zurich, 8057 Zurich, Switzerland; 3 Center for Applied Biotechnology and Molecular Medicine (CABMM), University of Zurich, 8057 Zurich, Switzerland

**Keywords:** Biomedical engineering, Bone, Bone, Biomechanics

## Abstract

Bone fractures commonly repair by forming a bridging structure called callus, which begins as soft tissue and gradually ossifies to restore rigidity to the bone. Virtual mechanical testing is a promising technique for image-based assessment of structural bone healing in both preclinical and clinical settings, but its accuracy depends on the validity of the material model used to assign tissue mechanical properties. The goal of this study was to develop a constitutive model for callus that captures the heterogeneity and biomechanical duality of the callus, which contains both soft tissue and woven bone. To achieve this, a large-scale optimization analysis was performed on 2363 variations of 3D finite element models derived from computed tomography (CT) scans of 33 osteotomized sheep under normal and delayed healing conditions. A piecewise material model was identified that produced high absolute agreement between virtual and physical tests by differentiating between soft and hard callus based on radiodensity. The results showed that the structural integrity of a healing long bone is conferred by an internal architecture of mineralized hard callus that is supported by interstitial soft tissue. These findings suggest that with appropriate material modeling, virtual mechanical testing is a reliable surrogate for physical biomechanical testing.

## Introduction

Long-bone fractures are among the most common and painful trauma-associated injuries of the musculoskeletal system^1,2^. Under conditions of relative stability, secondary fracture healing progresses by both intramembranous (periosteal) and endochondral bone formation to produce a structurally heterogeneous, functionally graded external bridging callus^3^. Most fractures heal within a few months, but in some patients, the process fails, resulting in a nonunion^4^. These patients have difficulty returning to work, report worse health-related quality of life than patients with AIDS, stroke, or type-I diabetes, and would give up 40% of their remaining lifespan to regain their health^5–7^. Nonunions remain a pervasive clinical problem in part because diagnosis currently requires subjective assessment of X-rays over long periods of watchful waiting, typically at least 6–9 months^8^. Unlike in animal models, clinical interpretation of the radiographic progress of fracture healing relies on a surgeon’s judgement of what can be seen, rather than an objective measurement of the structural mechanics of the bone^9^.

To address this clinical need, we previously developed a non-invasive, imaging-based virtual biomechanical test for assessing the progress of fracture healing. First, we developed a methodology for processing computed tomography (CT) scans to develop subject-specific 3D finite element models of healing bones^10,11^. In a recent ovine study, we demonstrated that a virtual torsion test outperforms subjective methods like radiographic scoring for predicting the progress of healing and that it is a reliable surrogate for postmortem physical torsion testing in intact tibiae^12^. In clinical pilot testing, out virtual torsion tests have been able to detect clinically significant delayed fracture healing in patients with comorbidities such as smoking and diabetes^13,14^.

**Figure 1:**
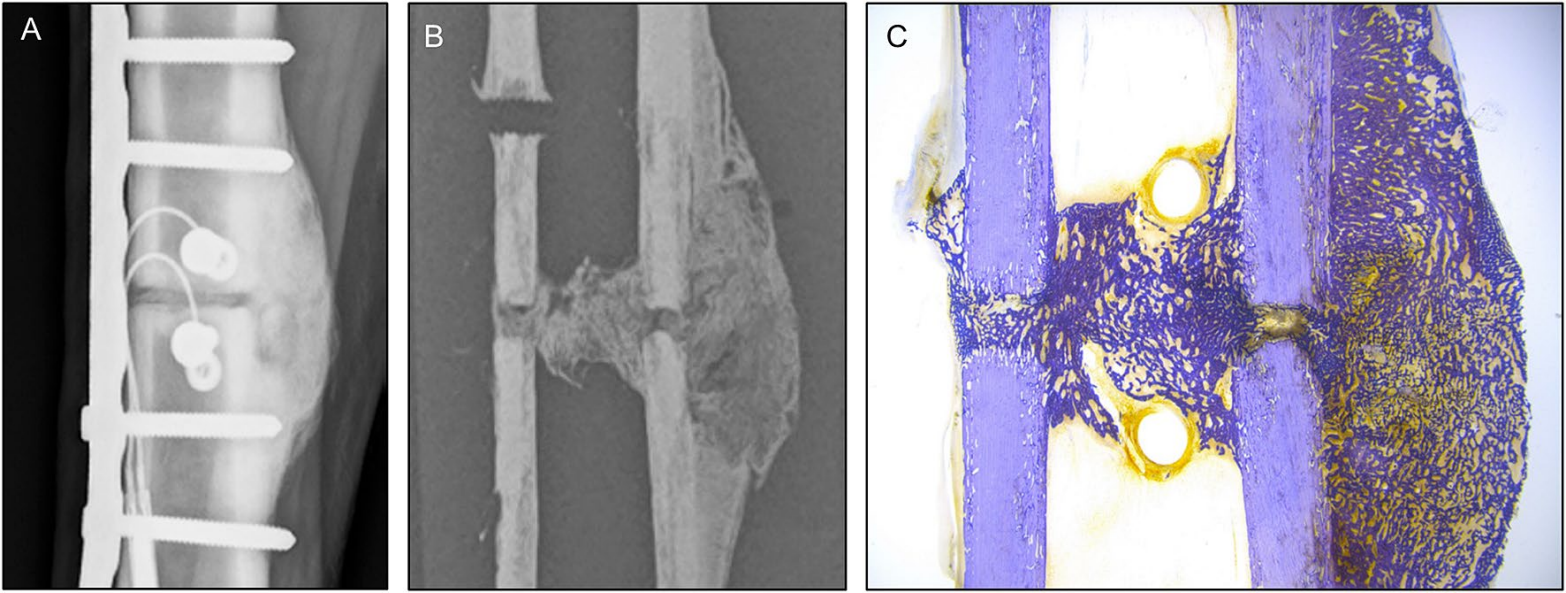
This case example taken from Schwarzenberg et al. (2021) shows a 3-mm osteotomy in an adult Swiss alpine sheep stabilized by medial plating at nine weeks post-op. (**A**) The large external callus completely bridged the fracture line. (**B**) A sectioned microradiograph illustrates heterogeneity within the callus, with highest density at the proximal and distal extents and lowest density near the fracture line and in the fracture gap. (**C**) Stained section (Toluidine Blue) shows denser, more mature woven bone away from the fracture line, and more porous soft callus in the central zone.

The key to success in virtual mechanical testing of bone mechanics is the use of density-dependent scaling laws for converting the radiodensity (gray value) in each voxel of the scan to a Young’s modulus, $E$^15–17^:

1 $$E=a{\rho }^{b}$$

where the coefficients $a$ and $b$ typically describe a linear or weak power-law relation and the radiodensity, $\rho$, is often expressed in either Hounsfield units [HU] or a calibrated bone mineral density using a radiological phantom. It is well understood that bone density is positively correlated with mechanical properties including Young’s modulus^18^. In subject-specific finite element models, elementwise material properties are mapped from the underlying scan data, such that regions of bone with lower density are assigned lower modulus values. This technique is highly effective for mimicking physical testing of intact bones but has a clear limitation when extended to be used in specimens with callus. Fracture callus is highly heterogeneous, as depicted in Fig. 1. This naturally adaptive structure develops in response to biological and mechanical cues and is comprised of hard callus (woven bone, newly mineralized or approaching the density of the intact cortex) and soft callus (fibrous and/or cartilaginous interstitial tissues)^19,20^. Our previous ovine study found that simply extending a density-modulus scaling law developed for cortical bone to include regions of callus produced strong correlations between virtual and physical biomechanical tests, but that the virtual tests over-predicted the measured torsional rigidity in osteotomized specimens by an average of 58%^12^. This over-prediction of rigidity when callus is present suggests that a constitutive material model derived for cortical bone does not completely capture the mechanical behavior of callus and that more work is needed to virtually replicate the in vivo biomechanics of healing fractures.

Accordingly, the purpose of this investigation was to develop a new constitutive mechanics model for fracture callus that captures its dual nature—hard and soft—and to test this method for assigning material properties in finite element models of healing ovine long bones. The hypothesis was that the virtual tests performed using the new dual-zone material assignment law for callus would outperform the virtual tests that use current single-equation methods when validated to physical biomechanical data.

## Results

### Effect of dual-zone material model on torsional rigidity

**Figure 2:**
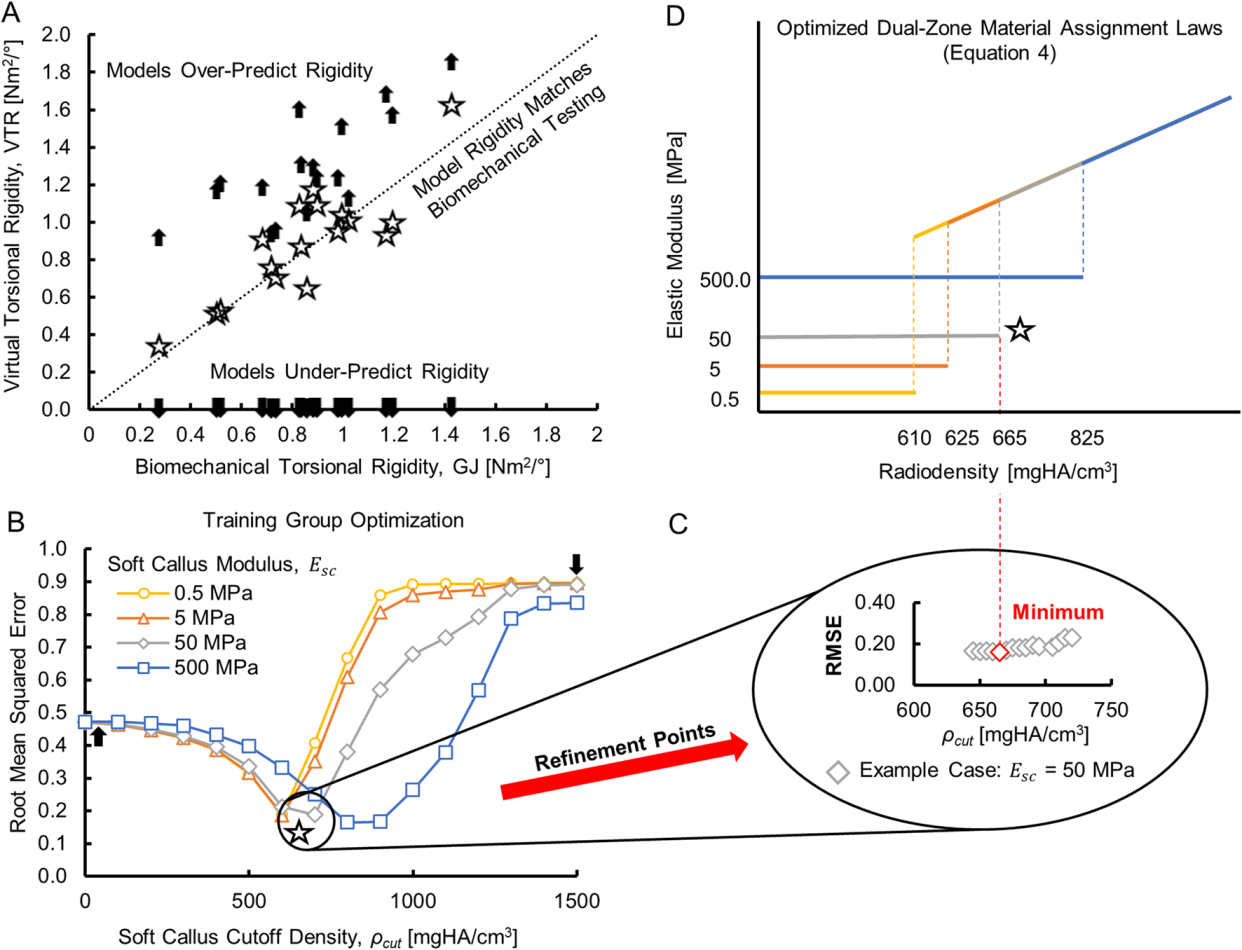
Snapshot of the optimization process and results. (**A**) Comparing the virtual and physical biomechanical tests for the N = 17 animals in the Training group showed that the single-zone material model (Eq. (3), up arrows) overpredicted the rigidity of osteotomized ovine tibiae. Two other representative examples of dual-zone material models were chosen to illustrate the results of optimization. For ${E}_{sc}$ = 50 MPa, the limiting case of maximum soft callus cutoff, ${\rho }_{cut}$ = 1500 mgHA/cm^3^, clearly underpredicted limb rigidity (down arrows). At the optimized cutoff value, ${\rho }_{cut}$ = 665 mgHA/cm^3^ (stars), the dual-zone material model out-performed the single-zone material model for predicting the biomechanical test results. (**B**) The approximate minimum RMSE between the virtual and biomechanical tests was identified for each ${E}_{sc}$ level by sweeping the ${\rho }_{cut}$ space. (**C**) Additional virtual tests were performed on refinement points about the initial minimum to identify the optimized ${\rho }_{cut}$ value (red diamond) for each ${E}_{sc}$ level. (**D**) The resulting four optimized combinations of soft callus modulus and density cutoff can be represented using the piecewise-defined dual-zone material model of Eq. (4).

Selected results from the material model optimization procedure are shown in Fig. 2. The reference single-zone material model (Eq. (3)) did not differentiate between soft and hard callus within the healing zone and it overpredicted the virtual torsional rigidity (VTR) of the whole structure (Fig. 2A, up arrows). In the dual-zone material model (Eq. (4)), any element with a calibrated radiodensity (${\rho }_{QCT}$) below the soft callus cutoff, ${\rho }_{cut}$, was assigned the soft callus modulus value (${E}_{sc}$ = 0.5, 5, 50, or 500 MPa). Within the optimization space, one limiting case was for ${\rho }_{cut}$ = 0 mgHA/cm^3^ for all ${E}_{sc}$ levels. In this case, the dual-zone material model performance was identical to the single-zone material model, with no elements assigned the soft callus modulus value and all elements treated as bone of varying density. Increasing ${\rho }_{cut}$ progressively increased the number of elements within the healing zone that were assigned the soft callus modulus. As ${\rho }_{cut}$ was increased, the virtual models transitioned from over-predicting to under-predicting rigidity compared to the biomechanical testing data. In the extreme case, increasing ${\rho }_{cut}$ into the range for cortical bone assigned the soft callus modulus to all elements in the model and resulted in very low torsional rigidity for the structure (Fig. 2A, down arrows). The optimization process was successful in identifying a value for ${\rho }_{cut}$ at each level of ${E}_{sc}$ that minimized the difference in torsional rigidity between the virtual and physical biomechanical tests (Fig. 2B–D). At the optimized combination of soft callus cutoff and modulus, the predicted VTR converged with biomechanical torsional rigidity (GJ) and the RMSE dropped to its minimum value (Fig. 2B,C). An interactive web application that illustrates the effect of changing ${\rho }_{cut}$ on the performance of the dual-zone material models can be found in Supplementary Fig. S1.

### Optimization results

**Figure 3:**
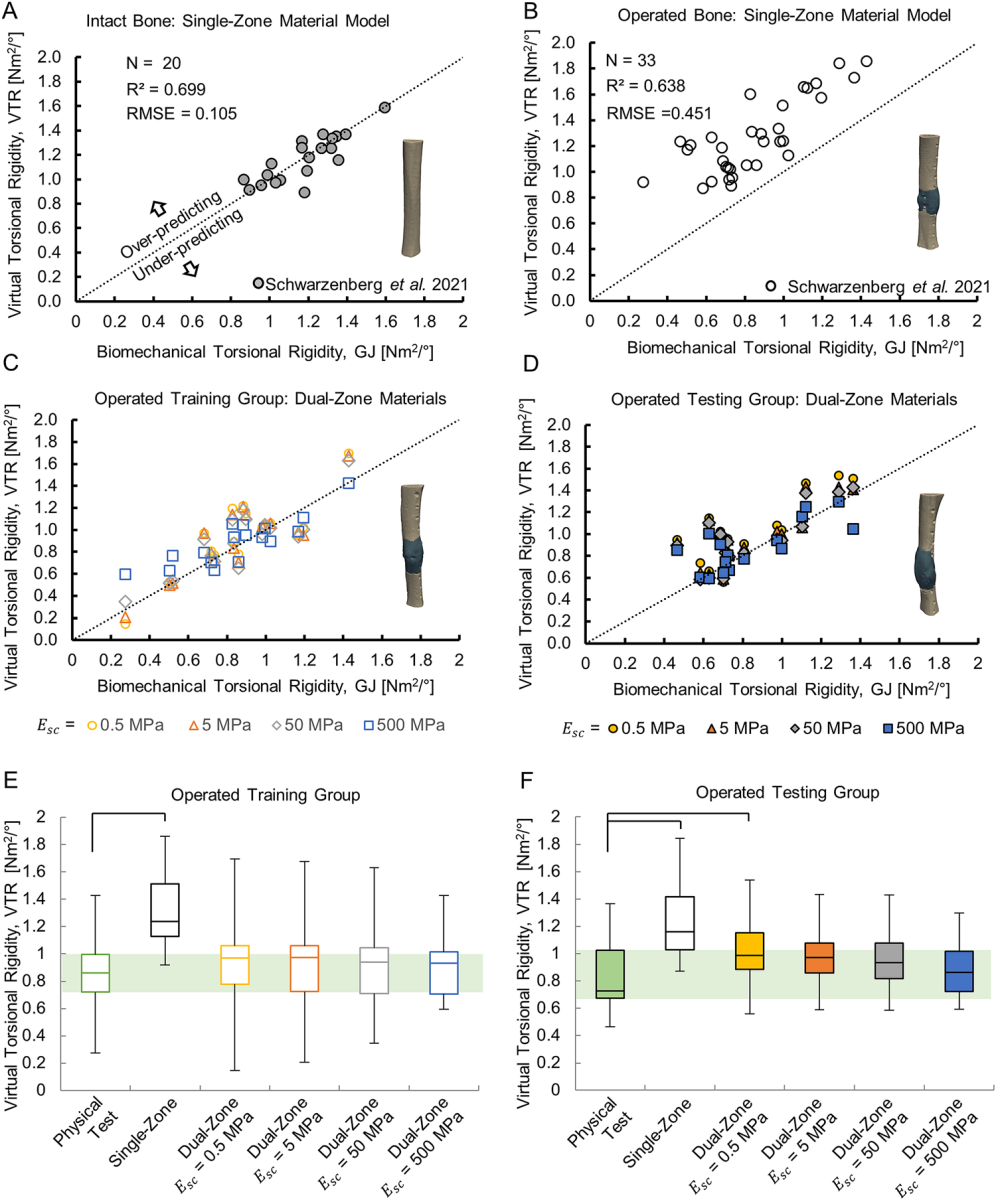
The results of the optimization. (**A**) The single-zone material model accurately predicted the stiffness of the intact tibiae (N = 20, R^2^ = 0.699, RMSE = 0.105). (**B**) The single-zone material model overpredicted the stiffness for fractured tibiae (N = 33, R^2^ = 0.638, RMSE = 0.451). (**C**) The material model optimized with the Training group data for each soft callus modulus value (0.5, 5, 50, 500 MPa) was shown to accurately predict stiffness for fractured tibiae (N = 17). (**D**) The optimized material model when applied to the Testing group was shown to accurately predict stiffness for fractured tibiae (N = 16). (**E**,**F**) Box plots comparing the single-zone and dual-zone material models showed that the optimized dual-zone model outperformed the single-zone model for fractured tibiae in both the Training and Testing groups. Bars show statistically significant group differences compared with biomechanical data.

Summary results of the optimization can be found in Fig. 3 and Table 1. The optimized density cutoffs for each soft callus modulus value (0.5, 5, 50, and 500 MPa) were determined to be 610, 625, 665, and 825 mgHA/cm^3^ respectively. The performance of the optimized dual-zone models at all four of the candidate soft callus modulus values is visualized in Fig. 3. The virtual mechanical tests with single-zone material assignment reliably recapitulated the physical mechanical tests for the intact ovine tibiae, with a strong correlation and good absolute agreement (Fig. 3A; R^2^ = 0.699, RMSE = 0.105). Applying the single-zone material model in the operated limbs also produced a strong correlation, but with VTR clearly over-predicting GJ (Fig. 3B; R^2^ = 0.638, RMSE = 0.451). The absolute agreement did not meet our defined criteria for success (RMSE < datum standard deviation (SD); see Table 2). In contrast, at all tested levels of soft callus modulus, the optimized dual-zone material models produced moderate-to-strong correlations between VTR and GJ with absolute agreement that met our defined success criteria for material model validation (all R^2^ ≥ 0.558 and RMSE ≤ 0.248 versus datum SD = 0.268; Fig. 3C,D and Tables 1 and 2). In both the Training and Testing groups, the optimized dual-zone model with ${E}_{sc}$ = 500 MPa had the lowest RMSE at 0.148 and 0.176, respectively. The model with ${E}_{sc}$ = 50 MPa had the second-lowest Training and Testing RMSEs at 0.160 and 0.203, respectively. Section contour plots showing a comparison of the material assignment distribution within the callus for the single-zone and optimized dual-zone material models have been included for representative animals in Supplementary Fig. S2.

**Table 1:** Performance of virtual models relative to biomechanical tests.

	Subgroup	RMSE between VTR and GJ	Pearson’s correlations for VTR vs. GJ	
			R^2^	significance
Single-zone material model	Intact (N = 20)	0.105	0.699	*p* < 0.001
	Operated (N = 33)	0.451	0.638	*p* < 0.001
Dual-zone material models: training group (N = 17)	${E}_{sc}$= 0.5 MPa	0.185	0.736	*p* < 0.001
	${E}_{sc}$= 5 MPa	0.178	0.724	*p* < 0.001
	${E}_{sc}$= 50 MPa	0.160	0.726	*p* < 0.001
	${E}_{sc}$= 500 MPa	0.148	0.726	*p* < 0.001
Dual-zone material models: testing group (N = 16)	${E}_{sc}$= 0.5 MPa	0.248	0.653	*p* < 0.001
	${E}_{sc}$= 5 MPa	0.222	0.605	*p* < 0.001
	${E}_{sc}$= 50 MPa	0.203	0.615	*p* < 0.001
	${E}_{sc}$= 500 MPa	0.176	0.558	*p* = 0.001

**Table 2:** Descriptive Statistics for Torsional Rigidity.

	Material model subgroup	Mean and standard deviation of torsional rigidity (VTR or GJ) [N-m^2^/°]
Biomechanical tests: all intact Tibiae (N = 20)	–	1.180 ± 0.187
Biomechanical tests: all operated animals (N = 33)	–	0.850 ± 0.268*
Biomechanical tests: training group (N = 17)	–	0.855 ± 0.279
Biomechanical tests: testing group (N = 16)	–	0.845 ± 0.264
Virtual Tests: Training group (N = 17)	0.5 MPa	0.924 ± 0.342
	5 MPa	0.910 ± 0.332
	50 MPa	0.899 ± 0.300
	500 MPa	0.892 ± 0.215
Virtual tests: testing group (N = 16)	0.5 MPa	1.028 ± 0.288
	5 MPa	0.987 ± 0.266
	50 MPa	0.956 ± 0.269
	500 MPa	0.884 ± 0.221

*Standard deviation of datum set for material model validation: 0.268.

Across both the Training and Testing groups, VTR predictions with the optimized dual-zone materials showed better agreement with GJ compared to the single-zone material model (Fig. 3E,F). All four of the candidate dual-zone material models had interquartile ranges that overlapped the interquartile range of the biomechanical data, while the single-zone law clearly overpredicted the stiffness. ANOVA post-hoc testing showed statistically significant differences between the single-zone VTR and biomechanical GJ for both the Training and Testing groups (both *p* < 0.001, Fig. 3E,F). There were no significant differences between biomechanical GJ and VTR for any dual-zone material model in the Training group. In the Testing group, there were no significant differences between GJ and VTR in the dual-zone models for soft callus modulus values of 5 MPa and higher; the only significant difference relative to the biomechanical test group was at the lowest soft callus modulus level (0.5 MPa; *p* = 0.007, Fig. 3F).

### Physiological performance evaluation: material properties, strain, and histology

For the Training group, all four of the optimized dual-zone material models produced correlation coefficients that were higher than that achieved with the single-zone material model (${R}^{2}$ ≥ 0.724 vs. ${R}^{2}$ = 0.638), with better absolute agreement with the physical testing (RMSE ≤ 0.185 vs. RMSE = 0.451). Correlation coefficients were slightly lower in the Testing group, but absolute agreement between VTR and GJ was still clearly improved relative to the single-zone material model. Except for the lowest soft callus modulus level (${E}_{sc}$ = 0.5 MPa), which was significantly different from the physical testing in the Testing group, the results did not provide a statistical justification for making a recommendation on the best-performing dual-zone material model (Fig. 3E,F and Table 1).

**Figure 4:**
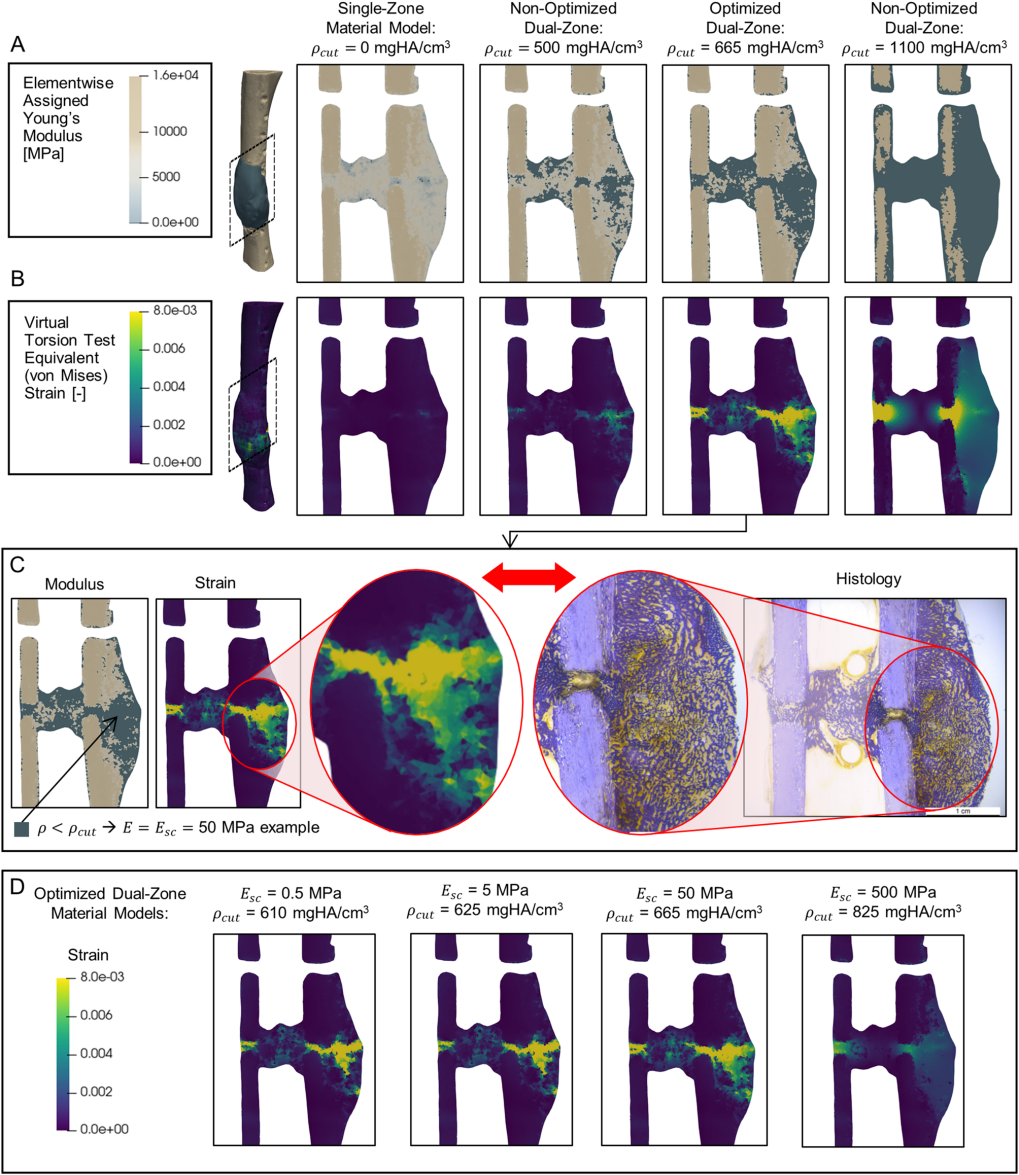
(**A**) Left to right: Coronal slice views of the finite element models showed that as the soft callus density cutoff, ${\rho }_{cut}$, was increased in the dual-zone model, larger regions of tissue within the callus were assigned the soft-callus modulus, ${E}_{sc}$. (**B**) Contour plots of equivalent (von Mises) strain showed that as ${\rho }_{cut}$ was increased, there were increasing strain concentrations in and around the fracture gap. (**C**) Qualitative comparison of strain contours to histological slices showed that at the optimized setting for the density cutoff (${\rho }_{cut}$ = 665 mgHA/cm^3^ for ${E}_{sc}$ = 50 MPa), the strain concentrations were colocalized with more porous, less-mineralized (i.e., softer) callus in the histological slice. (**D**) Comparing the strain contours across the four candidate optimized dual-zone material models, the three lowest ${E}_{sc}$ levels had patterns of strain concentration in and around the fracture line that reflected the observed localization of immature callus in the histological slices. Of these, ${E}_{sc}$ = 50 MPa had the lowest RMSE between virtual and physical tests (see Table 1).

To gain additional insights on possible differences in the structural model performance between these groups, contour plots of equivalent (von Mises) strain and Young’s modulus in the callus region were qualitatively compared to the histological sections. Example contour plots and section images are shown in Fig. 4 for one representative animal and each of the four candidate dual-zone material models (optimized combinations of ${E}_{sc}$ and ${\rho }_{cut}$). For low values of the density cutoff, ${\rho }_{cut}$, the callus region contained a large proportion of unmodified elements that were assigned material properties using the cortical bone scaling law (Eq. (3)), resulting in a stiffer structure with low strains throughout. Strain contour plots showed that increasing ${\rho }_{cut}$ was also associated with increasing concentrations of strain in and around the fracture gap. At high ${\rho }_{cut}$, the strain field became non-physical with poor correspondence to histological patterns. At the optimized combinations of ${E}_{sc}$ and ${\rho }_{cut},$ the assigned mechanical properties and strain contours were qualitatively well-matched with the localization of less-mature callus in the histological sections. Additional side-by-side comparisons of strain and histological slices can be found in Supplementary Fig. S3.

## Discussion

The development of a clinically relevant, reliable, scalable, non-invasive test to assess bone healing could have profound impacts on the care of nonunion fractures. Detecting problems in bone healing earlier would enable earlier intervention when warranted, and potentially better outcomes for the most at-risk patients. Image-based virtual mechanical testing shows promise for addressing this need, but one key barrier to clinical translation of this technique is the problem of material modeling in fracture callus. Callus is an inherently transient material that persists in a living organism for only a few months before being remodeled. Unlike native bone, the mechanical properties of callus cannot be studied in human cadavers, so our understanding of the material mechanics and structural organization of this tissue must be strongly informed by observations from large animals.

The results of this study clearly demonstrate that the material properties of the tissues in the callus region for ovine long bones are not monolithic and should therefore not be treated as such. This idea has strong resonance with histomorphometric data showing subzones of different composition within fracture callus and gradients in mineralization that develop in both space and time^21–23^. Vetter et al. (2010) performed quantitative tissue type mapping on sections from osteotomized ovine tibiae across the stages of early healing and found dramatic heterogeneity of density in regions where bone was forming, with intermixing of bony and fibrous tissues and a moving front of bone formation^24^. This agrees with nanoindentation mapping data that revealed local structural heterogeneity within small regions of callus and evidence that calcium deposition correlates with local tissue modulus^25^.

The dual-zone or piecewise material model adopted in this study helps to capture callus heterogeneity in two ways. First, it recognizes that where the callus contains bone, this tissue should be locally modeled as bone. To achieve this, we extended our previously validated scaling law for ovine cortical bone to include the radiodensity range of woven bone within the callus. Second, the dual zone material model acknowledges that in early or slow healing, a substantial portion of the healing zone may be comprised of fibrous and cartilaginous soft tissues. The results of this study confirm that from a structural mechanics perspective, soft tissues within the callus are not functioning simply as low-density bone. This can be inferred because using a bone-derived radiodensity scaling law to assign their mechanical properties leads to a systematic over-prediction of limb rigidity. In contrast, by dropping the assigned soft tissue modulus for low-density regions within the callus, strong correlations between the virtual and physical mechanical tests were achieved with greatly improved absolute agreement compared to a single-zone material model approach. It should be noted that the dual zone material model is not limited to a linear association between mineralized tissue density and modulus and a constant value to model soft callus tissue. The two zones of the material model are not required to be coupled and theoretically could be defined using any equations that are appropriate for the species and anatomic site of interest.

Importantly, these results were achieved using a purely objective, automatic density-based thresholding technique for identifying soft callus that does not require histological analysis, contrast enhancement, or user inputs to define tissue type boundaries. Both hard callus and cortical bone are treated using the same piecewise material law and do not need to be separately segmented. This approach has key advantages for preserving objectivity because detecting the boundaries between old cortical bone and the most-mature hard callus is very challenging using density-based thresholding^12^. In another study, we developed an image processing technique for assessing remodeling at the cortical-callus boundary in sheep. We found that during early healing, the cortical wall density decreases to create a smooth density gradient between the old and new bone^26^. This evidence supports our decision to treat cortical bone and hard callus using a single material assignment law and also highlights the potential challenges of pursuing an alternative tri-zone material formulation for cortical bone, hard callus, and soft callus. Development of a region-specific scaling law for woven bone that is distinct from cortical bone would require density-based segmentation that can differentiate between old and new bone. In our experience with sheep, remodeling obfuscates the cortical-callus boundary such that cortical segmentation cannot be performed exclusively through density-based methods and may require user inputs that are not repeatable. Theoretically, the same principle would also apply to the potential inclusion of trabecular bone, which was not present in the samples tested in this study but could require a distinct material model and the development of segmentation methods to account for mixed cortical-trabecular sites.

Considering all the results together, the data provide support for the hypothesis that virtual mechanical testing with a dual-zone material assignment law that captures both the hard and soft nature of callus would out-perform single-zone material modeling when validated to physical biomechanical data. Good absolute agreement was achieved between all virtual and physical tests in Training and Testing groups (RMSEs lower than datum SD). Correlations between the virtual and physical test results were slightly lower in the Testing group, but this was expected because the optimization was performed on the Training group. In order to rule out any dependency on grouping, a cross-validation of the optimization was performed. The Training and Testing groups were randomly resampled. The resampled Training group produced identical results to the original Training group (${\rho }_{cut}$ = 665 mgHA/cm^3^ at ${E}_{Sc}$= 50 MPa, Supplementary Fig. S5). Across all groupings considered, the dual-zone material model was superior to the single-zone model at predicting biomechanical tests and it met the defined criteria for success for this material model validation. Overall, this suggests that when implemented with a dual-zone material model that captures the soft/hard heterogeneity of tissues within the callus, virtual mechanical testing is a reliable surrogate for physical biomechanical testing of healing ovine long bones.

While the results of this study clearly show that modeling the dual soft-hard composition of the callus is necessary to achieve good absolute agreement with physical validation data, the data do not provide a strong statistical justification to guide the choice of soft callus modulus, ${E}_{sc}$. A literature review led to uncertainty regarding the true mechanical properties of fibrous and cartilaginous constituents of callus^27–33^, so a three orders-of-magnitude variation in soft callus modulus values was tested (${E}_{Sc}$ = 0.5, 5, 50, or 500 MPa). After optimization of the density cutoff for applying the soft modulus, we found that the dual-zone models with ${E}_{sc}$ ≥ 5 MPa met our criteria for success based on quantitative analysis of torsional rigidity. This finding is both a strength and a weakness of our approach. It suggests that the bony architecture within the callus contributes most of the structural integrity and that modeling is relatively insensitive to the choice of soft tissue modulus when the objective is to assess whole-bone rigidity. However, defining success this way comes at a price, which is uncertainty regarding the localization of strains inside the callus. Considering both the quantitative analysis of ${R}^{2}$ and RMSE and the qualitative comparisons of strain maps and histology, we recommend implementation of the dual-zone material model with a soft callus modulus, ${E}_{sc}$ = 50 MPa, for radiodensities below 665 mgHA/cm^3^. Further optimization of ${E}_{sc}$ to achieve quantitative agreement with physical tests based on strain would require data that was not available in our mechanical tests, such as the use of image-based strain mapping during mechanical testing of sectioned samples^29^. Unfortunately, these tests are destructive and have yet to be tried with a material-model optimization approach such as we have described, but a convergence of these methods merits further investigation.

This work is not without limitations. CT scanning without contrast enhancement does not provide the ability to differentiate between soft tissues based on their radiodensity, so we were not able to use imaging to separately define regions of cartilaginous and fibrous soft tissues. We have also employed a local (elementwise) isotropic linear elastic material model for all tissues in the dual-zone model. The use of linear elastic models for bone is widespread common practice in finite-element modeling of bone, but cortical bone exhibits anisotropy particularly with respect to its strength and post-yield behavior^34,35^ and the soft tissues present in early fracture healing can have viscoelastic or poroelastic behavior^36–39^. The mechanical testing data for this study used a constant loading rate (5°/min) and produced linear torque–angle response curves^12^. This observation lends support to the theory that the elastic bone contribution within the callus dominates the overall structural mechanics, at least at this stage of healing. Further research with non-destructive testing at multiple loading rates would be required to explore the suitability of piecewise nonlinear material models for representing these tissues.

Finally, the translational potential of these findings must be considered in the context of our choice of animal model. Our use of ovine data to develop the dual-zone material model is consistent with the widespread preference for sheep in osteotomy fracture models^40^ due to their similar bone healing rate and remodeling potential in comparison with humans^41–43^. However, sheep are not a perfect mechanical model for human bone. Ovine cortical bone is plexiform with few Haversian canals and its microstructure is more akin to woven bone than to human cortical bone^41^. This may have contributed to our success in using a single material scaling law to assign mechanical properties across the range of radiodensities including hard callus and old cortical bone. Hard or mature callus in humans may be more mechanically distinct from cortical bone than it is in sheep, but obtaining in vivo load-response data in healing human bones remains challenging, and this limitation may be difficult to overcome. Replicating the procedure we have reported here with osteotomies in other large animals could provide further evidence of generalizability between species and establish a framework for scaling the dual-zone material model for use in humans. However, all such studies would be limited by the fact that transverse osteotomies are a controllable but highly simplified representation of naturally occurring traumatic fractures. Surgical creation of more complicated geometries such as spiral fractures or comminuted fractures would add to the complexity of the study design and the difficulty of maintaining consistency across an already biologically diverse cohort of animals. Despite this limitation, the use of a dual-zone material model for callus in traumatic fractures would be justified based on the assumption that the tissues involved in secondary bone repair develop irrespective of the fracture morphology.

In conclusion, this work shows how careful modeling of material inhomogeneity within the callus region is required to accurately replicate the measured torsional rigidity of osteotomized ovine tibiae using virtual mechanical tests. Use of a single material assignment law developed for intact cortical bone overpredicts the rigidity of healing bones because it treats all tissue within the healing zone as bone of varying density. In contrast, a dual-zone approach that differentiates between soft and hard callus achieves strong correlations between virtual and physical test results with good absolute agreement. This material modeling method also reveals that much of the structural integrity of the healing bone is conferred from an internal architecture of mineralized hard callus with interstitial soft tissue. Overall, the results demonstrate the translational potential of image-based virtual mechanical testing as a reliable, non-invasive assessment of bone healing.

## Methods

### Animal study information

**Figure 5:**
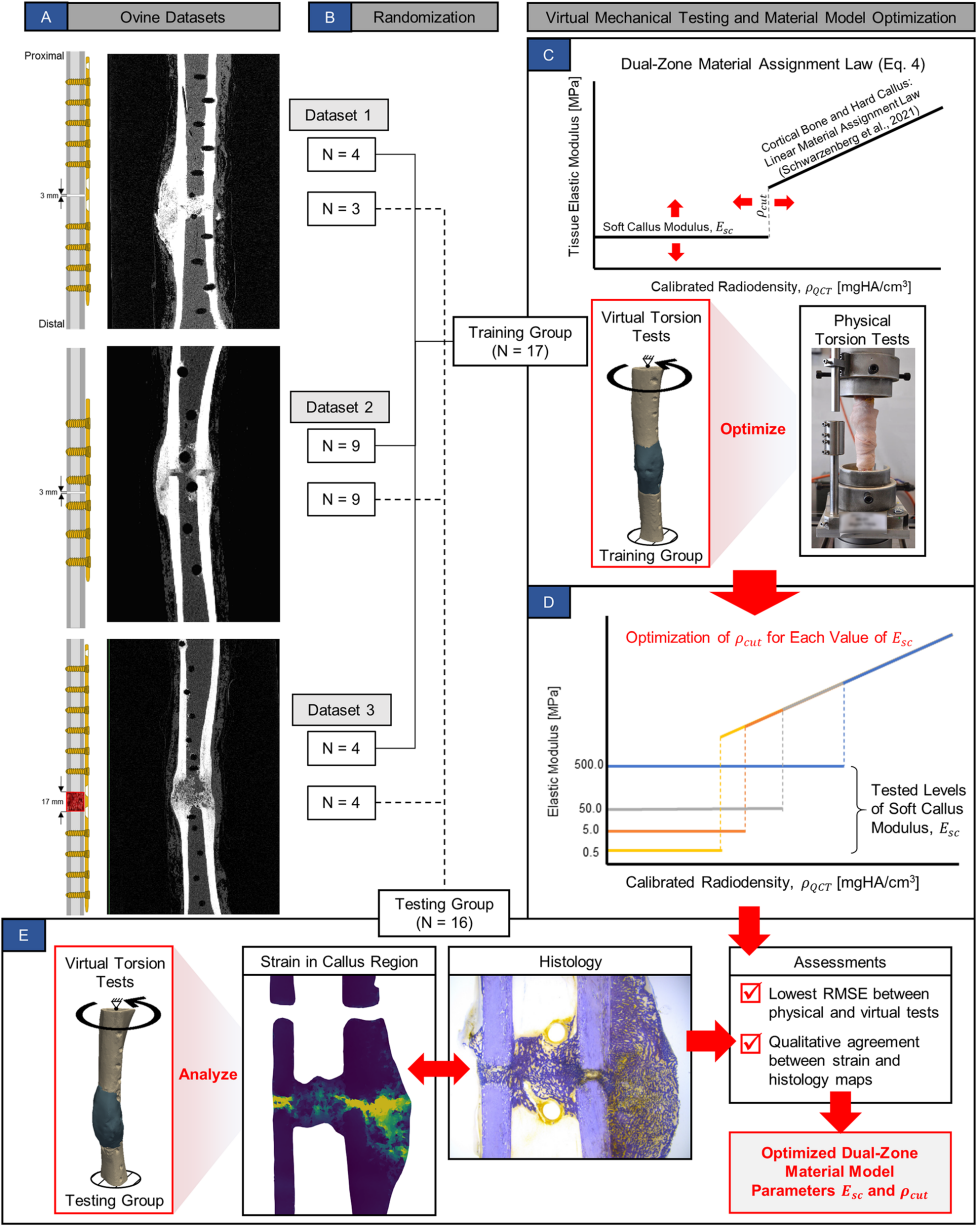
This flowchart outlines the entire material model optimization process. (**A**) In vivo data was derived from three previously completed ovine osteotomy studies, including two normal healing cohorts and one delayed healing cohort. A total of N = 33 animals had both µCT scans and postmortem biomechanical testing data available for analysis. (**B**) Animals were randomized into Training and Testing groups. (**C**) Virtual mechanical testing was performed on the Training group models and a response surface was constructed to optimize the selection of the soft callus modulus, ${E}_{sc}$, and density cutoff, ${\rho }_{cut}$. The objective of optimization was to minimize the RMSE between virtual and physical torsion tests. (**D**) Optimization produced four candidate piecewise material models (combinations of ${E}_{sc}$ and ${\rho }_{cut}$). (**E**) Material model performance was evaluated quantitatively based on RMSE relative to the physical testing data and qualitatively by comparison of predicted strain in the callus zone with histological slices for each animal.

Forty-four adult female Swiss alpine sheep (2–3 years old, weighing 59–87 kg) were part of multiple previously completed research studies with three different tibial osteotomy models stabilized by medial plating (Fig. 5A). Taken together, these animals comprised three experimental datasets across a wide spectrum of healing responses. Dataset 1 consisted of data from seven animals with a 3 mm gap defect stabilized with a 12‐hole stainless steel plate (broad straight veterinary 3.5 mm locking compression plate (LCP), 159 mm in length, with 3.5 mm bicortical screws; DePuy Synthes). Dataset 2 consisted of data from 18 animals with a 3 mm gap defect stabilized with a six‐hole titanium plate (broad 4.5/5.0 mm LCP, 115.8 mm in length, with 5 mm bicortical screws; DePuy Synthes). Dataset 3 consisted of data from eight animals with a 17 mm defect augmented with autografts and stabilized with a 13‐hole stainless steel plate (broad straight veterinary 3.5 mm LCP, 172 mm in length, with 3.5 mm bicortical screws; DePuy Synthes). The 3 mm defect models (Datasets 1 and 2) represented a noncritical size defect capable of spontaneously healing. Sheep in Datasets 1 and 2 were killed at 9 weeks. The 17 mm graft model (Dataset 3) represented a critical size defect that would not spontaneously heal without autograft augmentation. Sheep in Dataset 3 were killed 12 weeks after surgery. The animals were first stunned using a captive bolt, then eye reflexes were checked to ensure that stunning resulted in unconsciousness. Exsanguination was then performed by cutting the main blood vessels, resulting in the death of the animal from cerebral anoxia. Additional detailed animal information for these studies has been previously reported^12^. All animal experiments were conducted in accordance with ARRIVE guidelines and approved by the local governmental authorities of the canton of Zurich, Switzerland and conducted according to the Swiss laws of animal protection and welfare and approved by the local governmental veterinary authorities (license numbers ZH071/17 and ZH183/17)^12^.

### Imaging, mechanical testing, & scan processing

After sacrifice, the tibiae were dissected and soft tissue removed. Micro-Computed Tomography (µCT) scans were performed using an XtremeCT II Micro‐CT scanner (Scanco Medical AG) with an X‐ray voltage of 68 kVp and X‐ray current of 1470 µA. The resulting scans had an isotropic resolution of 60.7 µm. To convert from Hounsfield Units (HU) to calibrated radiodensity (${\rho }_{QCT}$, mgHA/cm^3^), a phantom was scanned with the same settings (Scanco KP70 phantom, QRM).

All samples were tested in a custom-made fixture using an Instron E10000 electrodynamic testing machine. Axial loading and torque were measured with a calibrated load cell (± 10 kN/ ± 100 Nm). The periosteum was stripped at the ends of each tibia, which were subsequently mounted into the custom fixture using Beracryl embedding medium. The periosteum was not stripped in the callus region. The diaphysis of each tibia was kept moist using saline‐soaked gauze. An axial preload of 5 N was maintained throughout the test. An internal rotation of 5° per minute was then applied. Biomechanical torsional rigidity was calculated as a linear regression of the loading curve between 6 and 10 N-m multiplied by the distance between the proximal and distal surfaces of the Beracryl pots (the gauge length).

The CT scans were processed using Materialize Mimics (21.0, Plymouth, MI) following a workflow developed for a previous study^10^. All µCT scans were first down-sampled to an isotropic resolution of 400 µm, which is comparable to clinical-resolution scanning. Cortical bone and callus regions were segmented to create anatomically representative virtual models of the biological samples. Isometric 3D surface views of all 33 osteotomized models are provided in Supplementary Fig. S4. After segmentation, the masks were united into a single surface model in preparation for volumetric discretization. To produce cohesive surfaces, the united models were wrapped with a gap closing distance of 1 mm and a smallest detail of 0.5 mm.

### Finite element modeling & virtual torsional rigidity testing

After scan processing, the finite element models were exported to 3-Matic (Mimics Innovation Suite) and a quadratic tetrahedral (Tet-10) mesh was applied to each model with a maximum surface edge length of 1 mm and a maximum interior edge length of 1 mm. The meshing procedure and a supporting mesh convergence study were previously described^10^. Elementwise material properties were applied to the finite element meshes based on underlying scan radiodensity data using a piecewise material assignment law further outlined within the *Material Model Definition* section below. Virtual torsional rigidity (VTR) testing was performed in ANSYS (2020 R2, Canonsburg, PA). The distal end of each tibia was fixed in translation and rotation and the proximal end was rotated by one degree about the long axis of the bone, leaving all other degrees of freedom unfixed. For each model, virtual torsional rigidity was calculated as follows:

2 $$VTR=\frac{ML}{\phi }$$

where $M$ is the calculated moment reaction, $L$ is the working length of the test segment, and $\phi$ is the applied angle of twist. Torsional rigidity was chosen over stiffness to eliminate the effects of varying specimen gage length across the cohort.

### Callus material model definition

Fracture callus is a heterogeneous, functionally graded composite structure comprised of a number of different tissue types, each of which have unique material properties^44^. The premise of our dual-zone material model is that the tissues around the fracture line can be mechanically represented as having two distinct constitutive components: hard callus (bony regions of varying density ranging from newly mineralized woven bone up to cortical bone) and soft callus (low-density fibrous and cartilaginous interstitial tissues). To accurately capture the structural mechanics of callus, it is necessary to treat these regions differently in the material model. To accomplished this, we defined a piecewise (dual-zone) material formulation that used a to-be-determined radiodensity threshold cutoff to differentiate between hard callus and soft callus. Above the cutoff, bone of varying density was represented using a species-specific density-modulus relation. Previously, we used an optimization technique to derive a scaling equation for the density-dependent Young’s modulus of ovine cortical bone and achieved close correspondence between physical and virtual torsion tests of intact ovine tibiae^12^:

3 $$E= 10225{ \times \left({\rho }_{QCT}\right)}^{1.0}$$

where *E* is Young’s modulus [MPa] and ${\rho }_{QCT}$ is the phantom-calibrated radiodensity [mgHA/cm^3^]. In the dual-zone material model, this material assignment law for cortical bone was simply extended to model the mechanical properties of all material (cortical bone and hard callus) above the density cutoff threshold. A Poisson ratio 0.3 was assumed for all elements^45,46^.

To extend the single-zone material model of Eq. (3) to a dual-zone piecewise application, two more parameters were required: a soft callus density cutoff (${\rho }_{cut}$) and a soft callus modulus value (${E}_{sc}$). The density cutoff value was used to specify the threshold below which elements were insufficiently mineralized to be treated as bone and should instead be modeled as soft tissue with modulus ${E}_{sc}$. Below the cutoff, the soft tissues within the callus were treated as homogeneous with a modulus much lower than that of bone. After a literature search, a definitive value for ${E}_{sc}$ could not be identified. However, upper and lower limits were identified based on previous work^27–33^. The candidate soft callus modulus values were therefore chosen on a logarithmic range to be ${E}_{sc}=$ 0.5, 5, 50, and 500 MPa. These values were tested in combination with a range of ${\rho }_{cut}$ density cutoffs via the procedure described in material model optimization below to determine the piecewise material formulation best able to replicate in vitro biomechanical testing.

Our density-delineated zonal approach to modeling callus was inspired by Mora-Macías et al*.* (2019), who used threshold-based segmentation to apply homogeneous mechanical properties within zones labelled as cortical bone, hard callus, and soft tissue^32^. Their approach was adapted from earlier work by Shefelbine et al*.* (2005), who postulated a dual-zone piecewise approach for modeling material properties in murine fracture callus^33^. We sought to combine the advantageous features of both methods (density-dependent mechanical properties of bone, density-based delineation between hard and soft callus) and seek a parameter set that would achieve close correspondence between physical and virtual mechanical tests of osteotomized ovine tibiae.

### Material model optimization

To perform parameter optimization for the dual-zone material model, the N = 33 operated tibiae were randomly assigned to a Training group (N = 17) and a Testing group (N = 16) using permutations generated in MATLAB (Fig. 5B). Animals from the three experimental datasets were randomized within group to the Training and Testing sets to maintain equal representation from the different surgical models. The optimization objective was to minimize the difference between calculated VTR and measured biomechanical torsional rigidity by varying ${\rho }_{cut}$ and ${E}_{sc}$ for all 17 Training group specimens simultaneously. The resulting response surface was evaluated based on root mean squared error (RMSE) between the virtual and physical torsional rigidities (Fig. 5C,D). A quantitative analysis of ${R}^{2}$ and RMSE and qualitative comparisons of strain maps and histology were considered to select a dual-zone material model (Fig. 5E).

To assign material properties to an individual model for a candidate parameter set (combination of ${\rho }_{cut}$ and ${E}_{Sc}$), first the raw material data for each element in the mesh was pre-processed using a custom function developed in MATLAB. In each finite element within the mesh, the gray value density in Hounsfield Units was converted to calibrated radiodensity (${\rho }_{QCT}$, mgHA/cm^3^) using information from a calibration phantom scan. For each element, if ${\rho }_{QCT}$ fell below the soft callus cutoff ${\rho }_{cut}$, it was assigned a soft callus modulus value (${E}_{sc}$ = 0.5, 5, 50, or 500 MPa, as described above). If ${\rho }_{QCT}$ for an element was above ${\rho }_{cut}$, it was treated as bone and assigned a modulus using Eq. (3). The piecewise function can be represented as follows:

4 $$E=\left\{\begin{array}{cc}{E}_{sc}& {\rho }_{QCT}<{\rho }_{cut}\\ 10225{ \times \left({\rho }_{QCT}\right)}^{1.0}& {\rho }_{QCT}\ge {\rho }_{cut}\end{array}\right.$$

To construct the response surface for optimization, testing was performed for each soft callus modulus value (0.5, 5, 50, and 500 MPa) with candidate values for the soft callus cutoff swept from ${\rho }_{cut}$ = 0 to 1500 mgHA/cm^3^ in increments of 100 mgHA/cm^3^, resulting in 16 tested levels for the soft callus cutoff (Fig. 2B). At each candidate combination of ${E}_{sc}$ and ${\rho }_{cut}$, the root-mean-square error (RMSE) between VTR and biomechanical torsional rigidity GJ was calculated based on all 17 Training group samples. Across the range of ${\rho }_{cut}$ values, the approximate minimum RMSE was then identified for each soft callus modulus (0.5, 5, 50, and 500 MPa). The virtual torsion tests were then run on the Training group models at additional ${\rho }_{cut}$ refinement points on either side of the approximate minima in increments of 5 mgHA/cm^3^ (Fig. 2C). After refinement, the RMSE minima were identified for each of the four candidate ${E}_{sc}$ values and the four corresponding optimized ${\rho }_{cut}$ values were recorded (Fig. 2D). Altogether, construction of the refined response surface for all models in the Training group required a total of 2363 virtual mechanical tests. All simulations were run on nodes of the High-Performance Computing (HPC) server hosted on Lehigh University’s campus and required a total of approximately 5,000 CPU-hrs.

To test the validity of the optimized piecewise material model, each of the four optimized soft callus cutoffs were then applied to the N = 16 Testing group models. The RMSE for VTR versus GJ was then calculated at each of the optimized combinations of ${E}_{sc}$ and ${\rho }_{cut}$ and the structural simulations results were saved for performance comparison between each of these four states.

### Data processing, visualization, and statistics methods

Simulation post-processing was performed in MATLAB. Mesh data, material model data, and the mechanical testing results from ANSYS were queried using custom MATLAB functions. Contour slice plots and 3D visualizations of models and results were generated using Paraview (5.9.0, Sandia National Laboratories, Kitware Inc, Los Alamos National Laboratory).

Descriptive statistics were generated using Microsoft Excel. Additional statistical analyses were generated using IBM SPSS Statistics 27 (Armonk, NY). Pearson’s correlations were used to determine the strength of association between virtual and biomechanical torsional rigidity datasets. A correlation coefficient $R$ ≥ 0.8 (${R}^{2}$ ≥ 0.64) was defined as *strong* and $R$ ≥ 0.6 (${R}^{2}$ ≥ 0.36) was defined as *moderate*^47^. The criteria for evaluating “good” absolute agreement between correlated measures was the requirement that root mean squared error (RMSE) be less than the standard deviation of the datum set. One-way repeated-measures ANOVA was performed on both the Training and Testing groups to determine if there were statistically significant differences between the measured GJ from biomechanical testing and predicted VTR in each of the four optimized material models (combinations of ${E}_{sc}$ and ${\rho }_{cut}$). Post hoc analysis with a Bonferroni adjustment was performed on pairwise comparisons to identify statistically significant differences. All values reported are averages and standard deviations unless otherwise stated. The statistical significance limit was *p* = 0.05.

## Supplementary Information

Supplementary Information.
