# Supplementary material for: Biomechanical duality of fracture healing captured using virtual mechanical testing and validated in ovine bones
Source: Sci Rep. 2022 Feb 15;12:2492. doi: 10.1038/s41598-022-06267-8 (PMC8847550; doi:10.1038/s41598-022-06267-8)
Supplement: Supplementary file 1 — Supplementary Information. [file 41598_2022_6267_MOESM1_ESM.pdf]

Biomechanical duality of fracture healing captured using virtual mechanical testing and validated in ovine bones

Brendan Inglis[a*], Peter Schwarzenberg[a], Karina Klein[b], Brigitte von Rechenberg[b,c], Salim Darwiche[b,c], Hannah L. Dailey[a*]

[a] Department of Mechanical Engineering and Mechanics, Lehigh University, Bethlehem, PA 18015

[b] Musculoskeletal Research Unit (MSRU), Vetsuisse Faculty, University of Zurich, 8057 Zurich, Switzerland

[c] Center for Applied Biotechnology and Molecular Medicine (CABMM), University of Zurich, 8057 Zurich, Switzerland

Corresponding Authors:

Brendan Inglis
27 Memorial Drive West
Bethlehem, PA 18015
1-610-888-6824
bji219@lehigh.edu

Hannah Dailey, PhD
19 Memorial Drive West
Packard Lab Room 356
Bethlehem, PA 18015
+1 (610) 758-4112
hannah.dailey@lehigh.edu

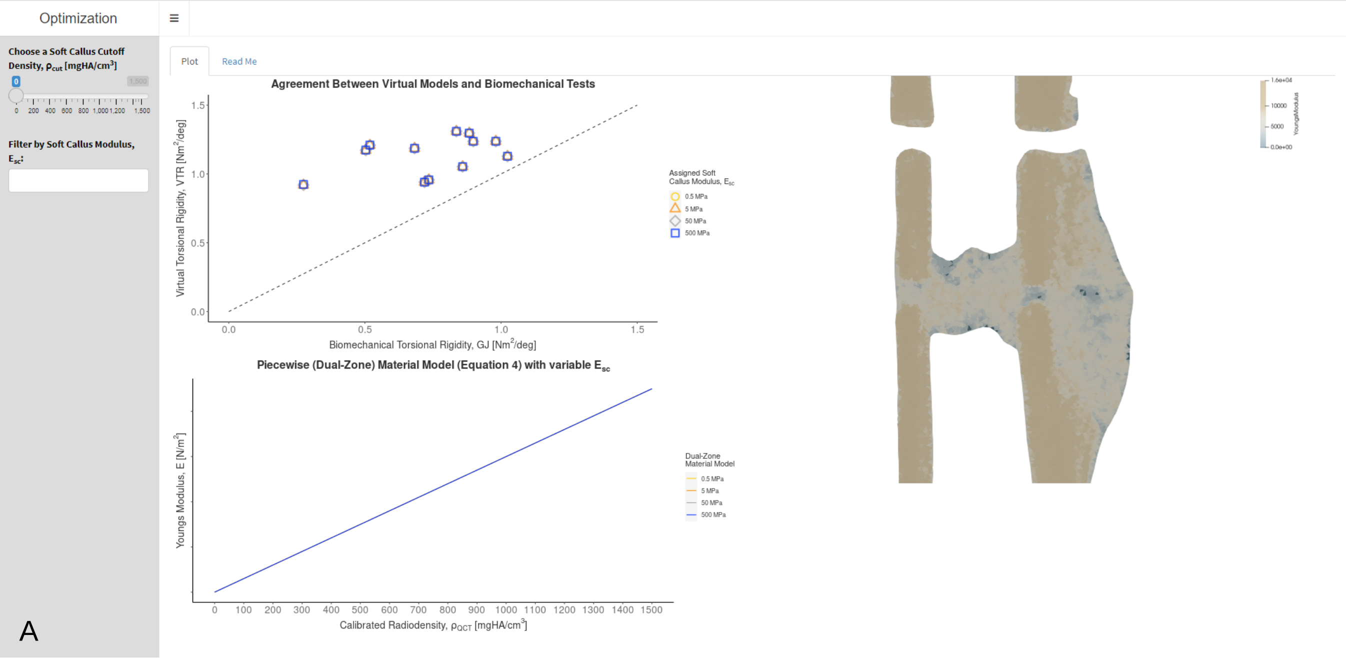

B **Interactive Optimization Shiny-App (Control-click)**

Supplementary Fig. S1. A) Preview of interactive web application. B) Embedded link to the interactive web application (control-click the bold text to open).

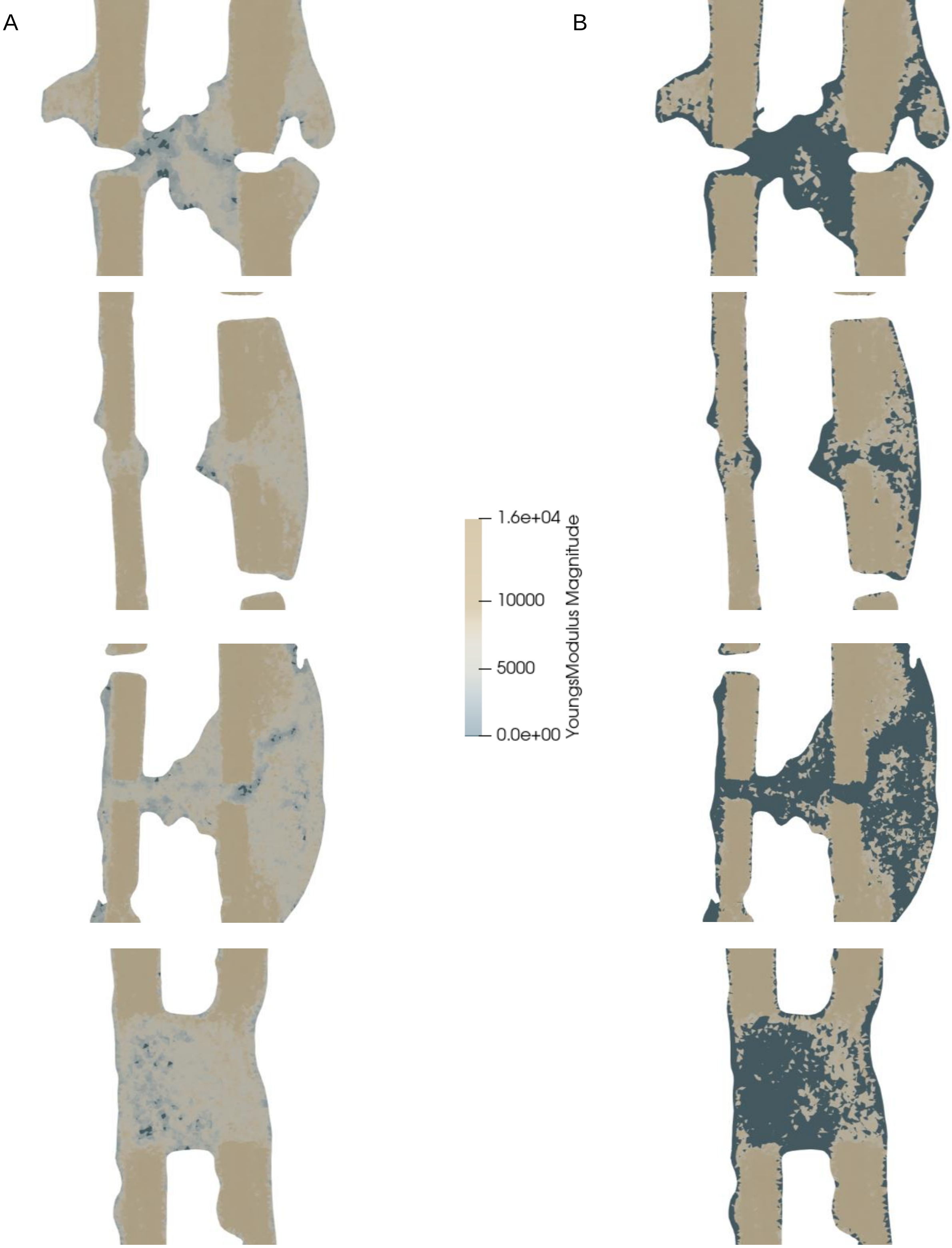

Supplementary Fig. S2. A) Select cross sections depicting material property distribution in single-zone material model. B) Corresponding cross sections with optimized dual-zone material model.

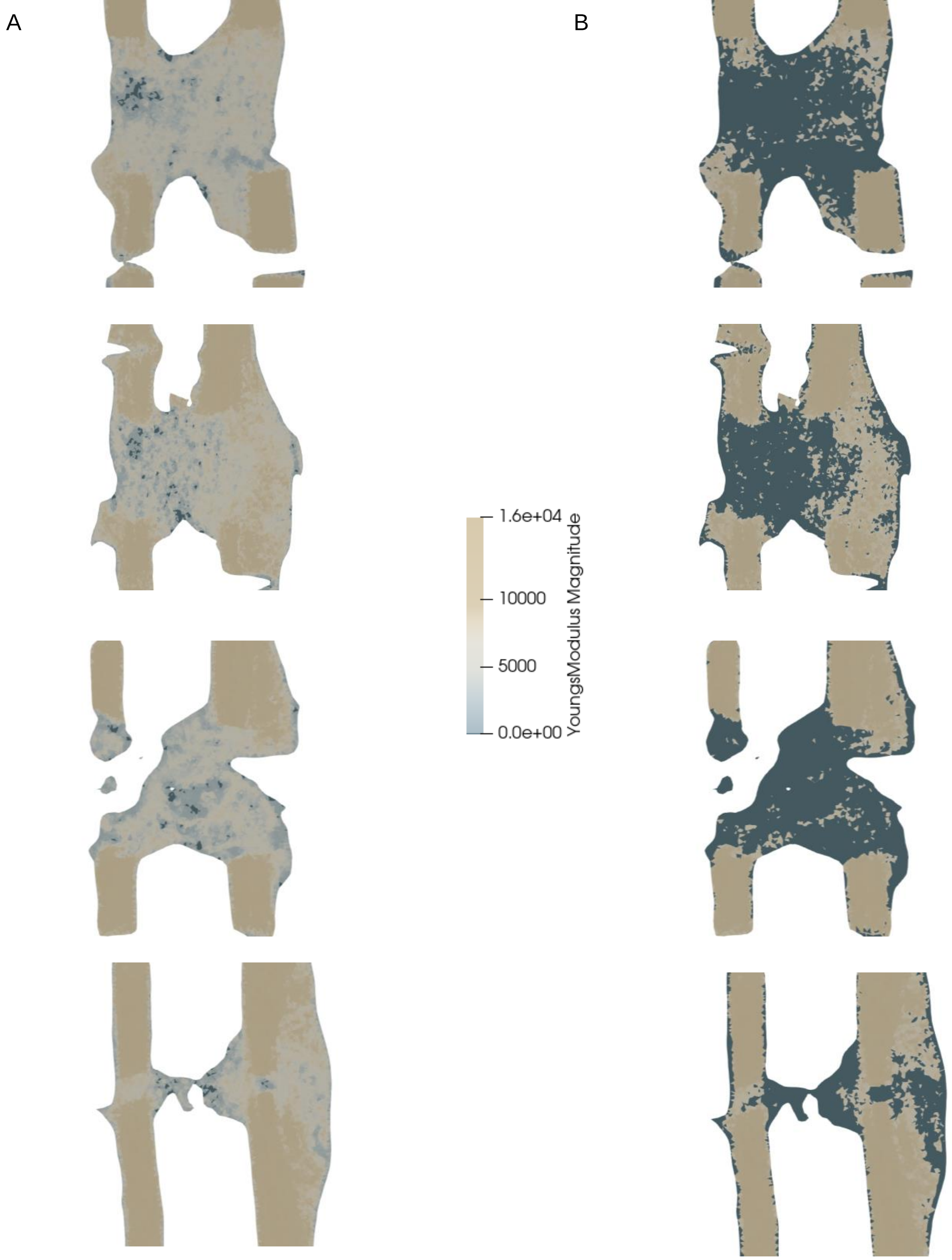

Supplementary Fig. S2. Continued

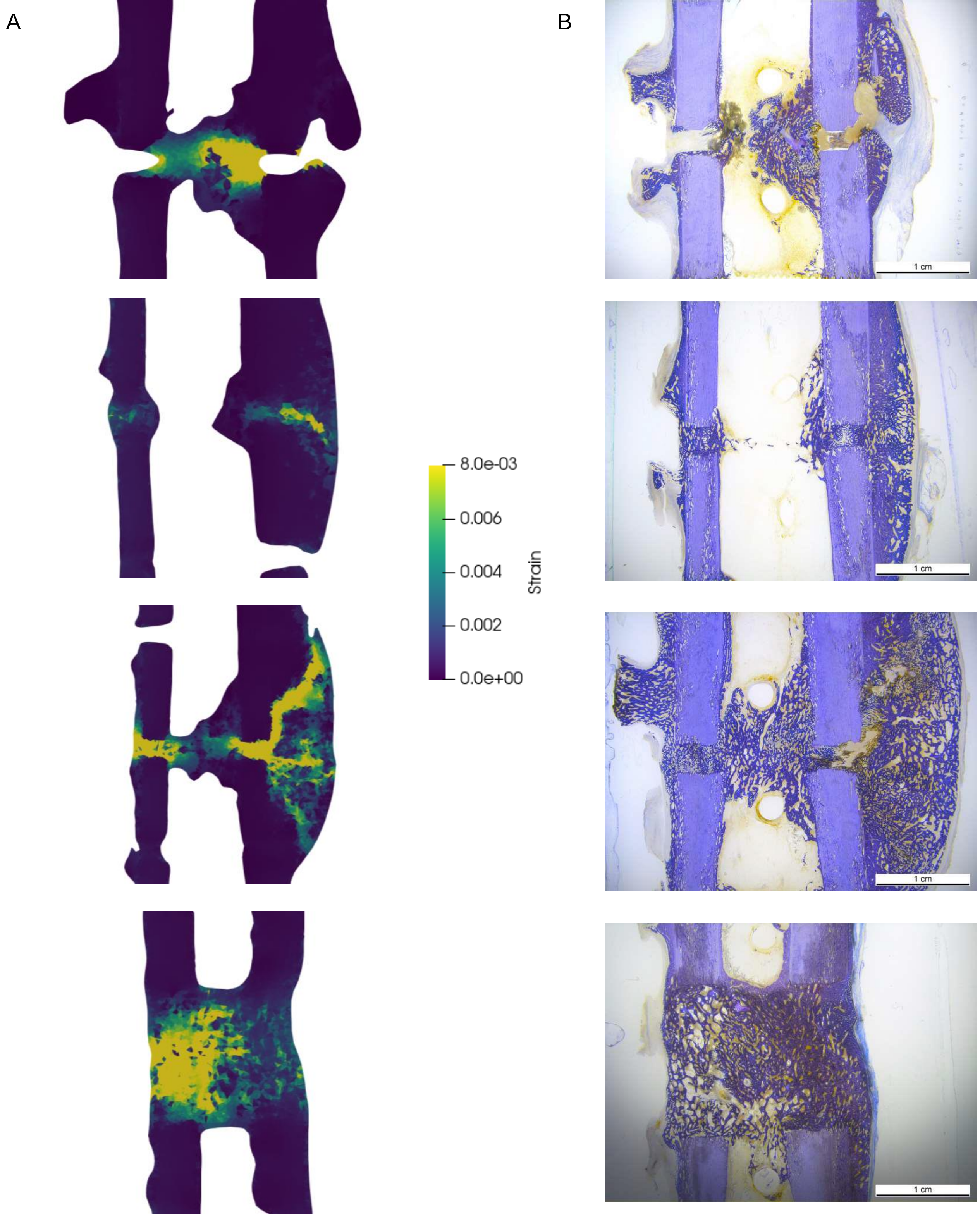

Supplementary Fig. S3. A) Select cross sections displaying the strain field within the callus region for the optimized dual-zone model. B) Corresponding biological histology slices.

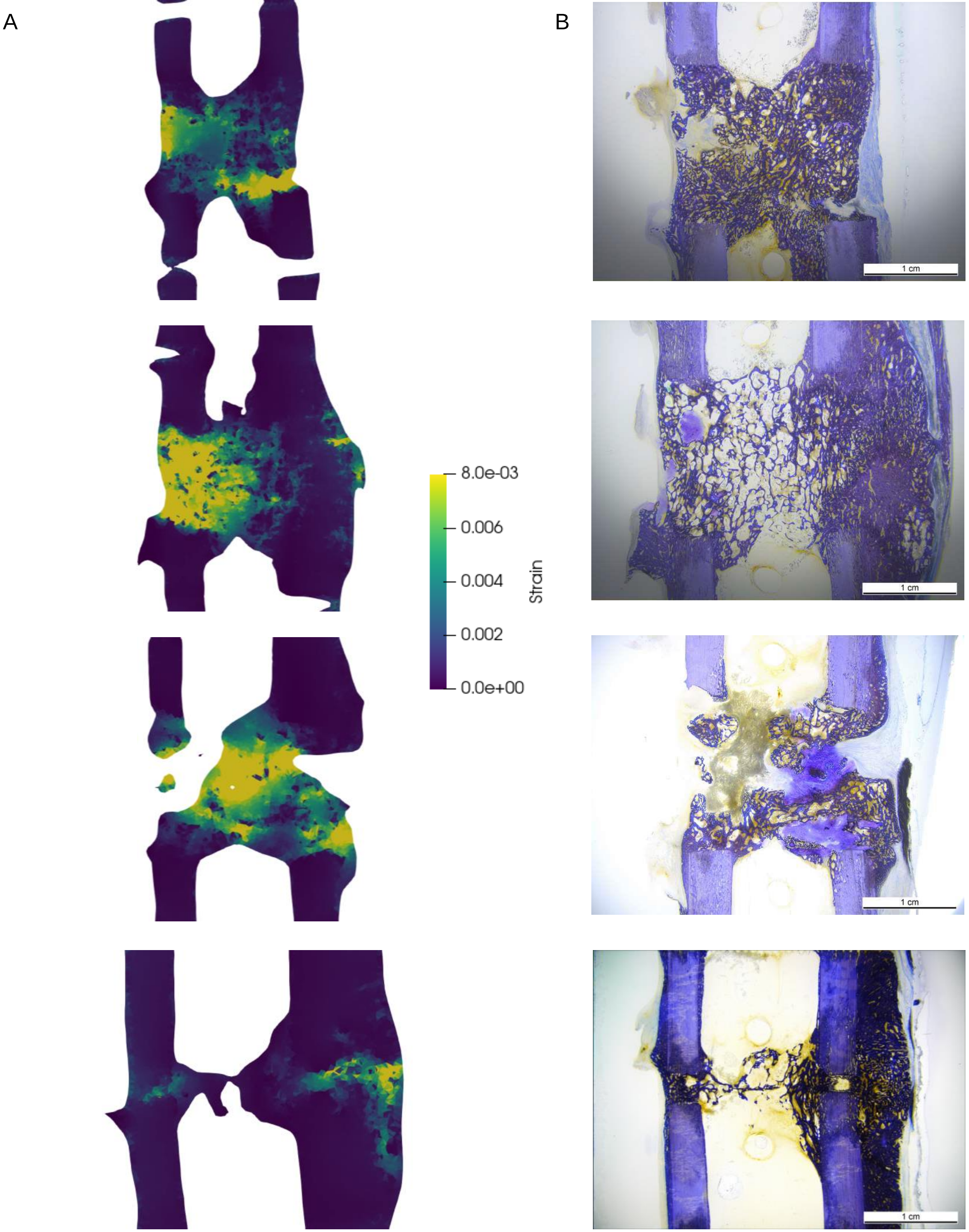

Supplementary Fig. S3. Continued

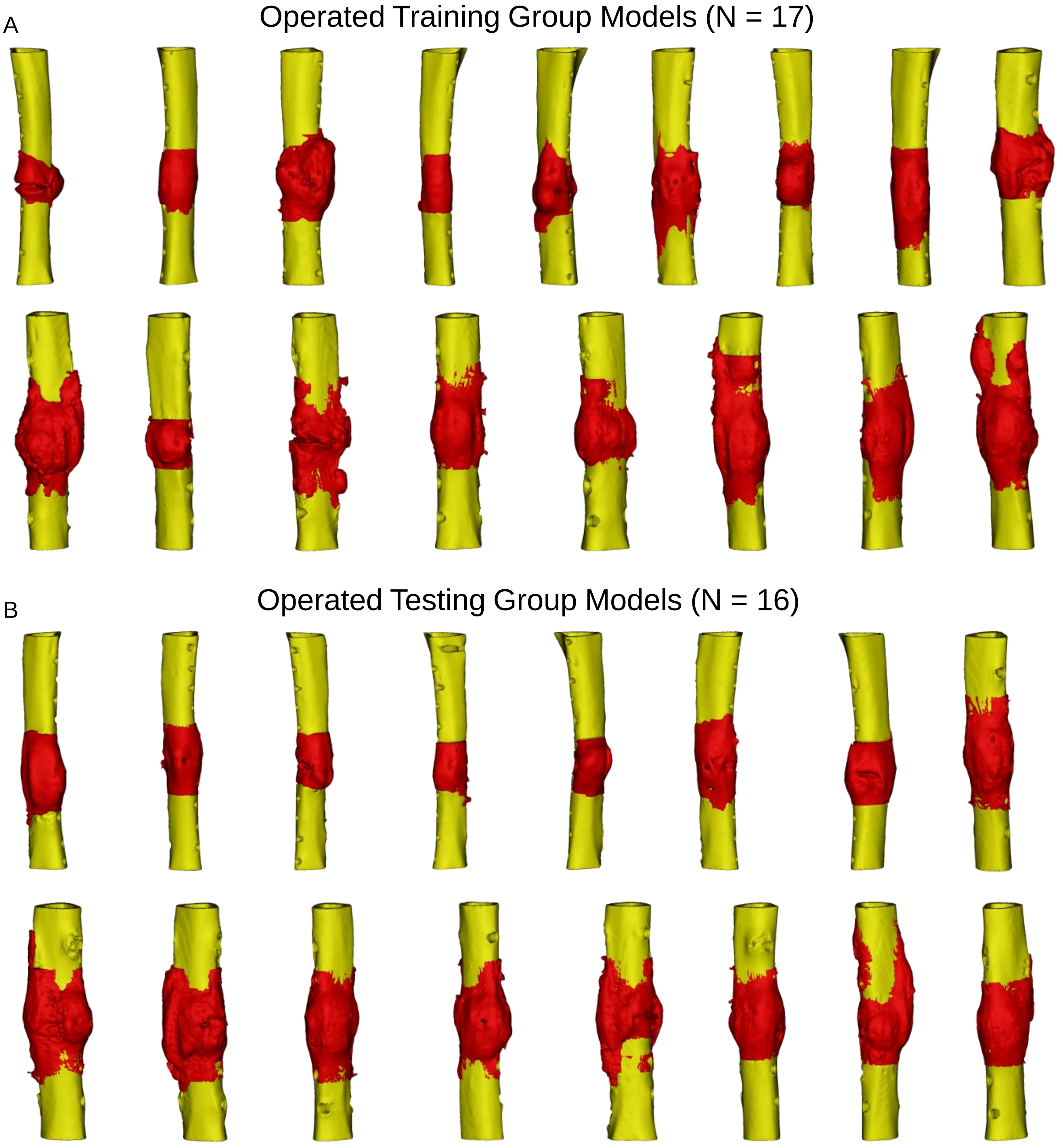

Supplementary Fig. S4. A) All 3D Finite Element Models in the Training Group. B) All models in the Testing Group.

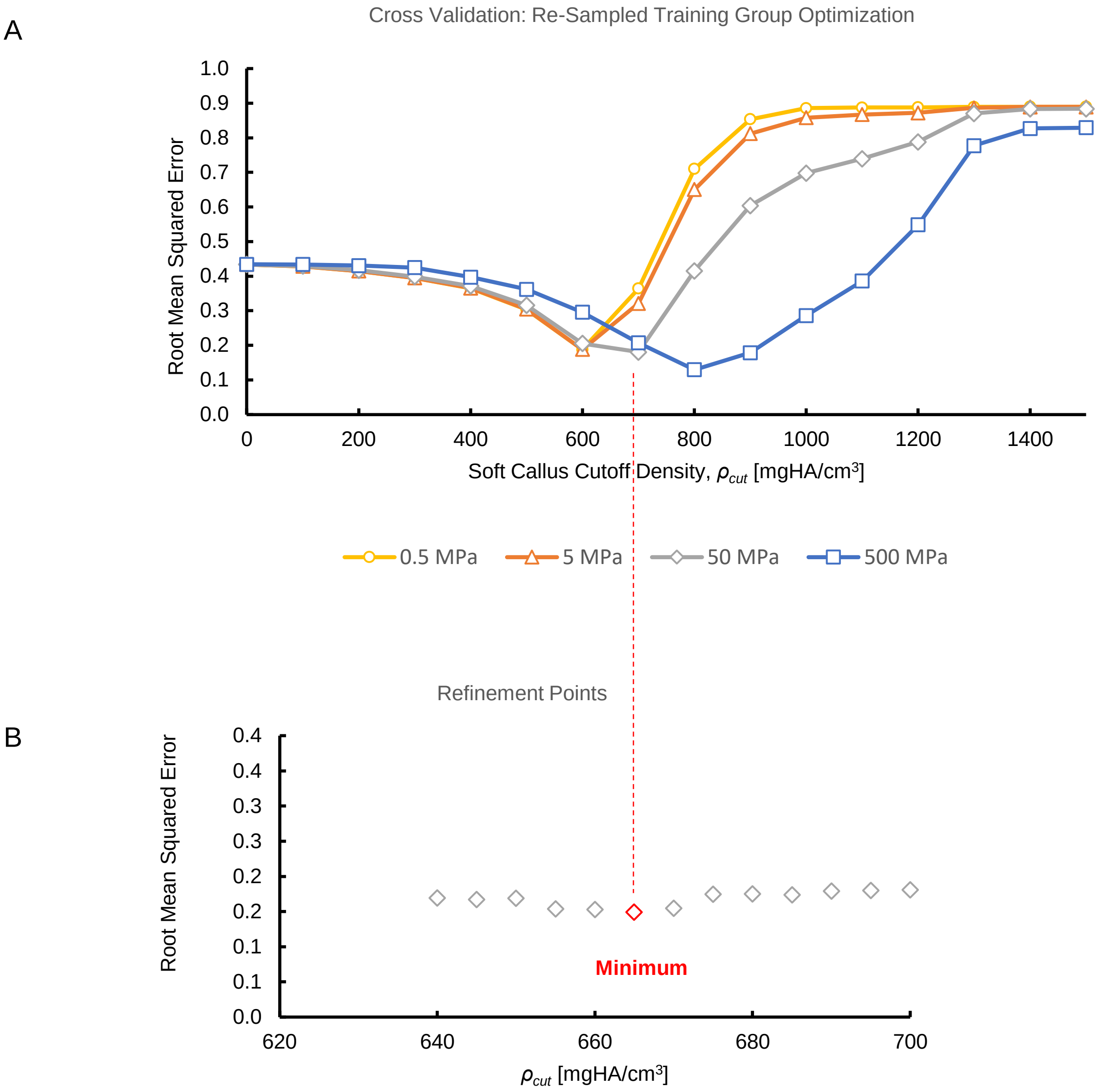

Supplementary Fig. S5. A) To determine the robustness of the dual zone method, a cross-validation was performed to test the result of optimization. A new random permutation of all 33 animals was used to generate the 16 animals that were assigned for the cross-validation. Using these 16 animals, the density cutoff range was swept in 100 mgHA/cm^3 increments. B) For the E_{sc} = 50 MPa soft callus modulus, the cutoff was refined in increments of 5 mgHA/cm^3. The resulting optimized density cutoff of 665 mgHA/cm^3 was identical to that of the original Training group. The RMSE in the cross-validation (0.149) was lower than that in the original Training group (0.160).
